# Sphere-forming cell subpopulations with cancer stem cell properties in human hepatoma cell lines

**DOI:** 10.1186/1471-230X-11-71

**Published:** 2011-06-14

**Authors:** Lu Cao, Yanming Zhou, Beibei Zhai, Jian Liao, Wen Xu, Ruixiu Zhang, Jing Li, Yu Zhang, Lei Chen, Haihua Qian, Mengchao Wu, Zhengfeng Yin

**Affiliations:** 1 Department of Molecular Oncology, Eastern Hepatobiliary Surgery Hospital, Second Military Medical University, Shanghai, China; 2 Department of Hepato-Biliary-Pancreato-Vascular Surgery, the First affiliated Hospital of Xiamen University, Xiamen, China

## Abstract

**Background:**

Cancer stem cells (CSCs) are regarded as the cause of tumor formation and recurrence. The isolation and identification of CSCs could help to develop novel therapeutic strategies specifically targeting CSCs.

**Methods:**

Human hepatoma cell lines were plated in stem cell conditioned culture system allowed for sphere forming. To evaluate the stemness characteristics of spheres, the self-renewal, proliferation, chemoresistance, tumorigenicity of the PLC/PRF/5 sphere-forming cells, and the expression levels of stem cell related proteins in the PLC/PRF/5 sphere-forming cells were assessed, comparing with the parental cells. The stem cell RT-PCR array was performed to further explore the biological properties of liver CSCs.

**Results:**

The PLC/PRF/5, MHCC97H and HepG2 cells could form clonal nonadherent 3-D spheres and be serially passaged. The PLC/PRF/5 sphere-forming cells possessed a key criteria that define CSCs: persistent self-renewal, extensive proliferation, drug resistance, overexpression of liver CSCs related proteins (Oct3/4, OV6, EpCAM, CD133 and CD44). Even 500 sphere-forming cells were able to form tumors in NOD/SCID mice, and the tumor initiating capability was not decreased when spheres were passaged. Besides, downstream proteins DTX1 and Ep300 of the CSL (CBF1 in humans, Suppressor of hairless in Drosophila and LAG1 in C. elegans) -independent Notch signaling pathway were highly expressed in the spheres, and a gamma-secretase inhibitor MRK003 could significantly inhibit the sphere formation ability.

**Conclusions:**

Nonadherent tumor spheres from hepatoma cell lines cultured in stem cell conditioned medium possess liver CSC properties, and the CSL-independent Notch signaling pathway may play a role in liver CSCs.

## Background

Hepatocellular carcinoma (HCC) is the fifth most common cancer and the third leading cause of cancer death worldwide [1,2]. Its overall incidence remains alarmingly high in the developing countries and is steadily rising across most of the developed countries [1-3]. Although the cytologic pathogenesis of HCC remains unclear, it has been proposed that only a small fraction of cancer cells with stem cell properties, named cancer stem cells (CSCs), is responsible for the initiation, progression, local and distant recurrence/metastasis of HCC, also for the failure of chemo- and radiotherapy. The clinical corollary of this hypothesis has been extended to proposals to treat cancer by targeting the putative liver CSCs.

Numerous attempts have been made to identify cells with stem cell properties in established HCC cell lines. Different research groups have reported that liver CSC fractions could be successfully enriched by some cell surface phenotypes, specifically CD133, CD90, CD44, epithelial cell adhesion molecule (EpCAM), OV6 and CD13 [4-9]. Nevertheless, these proteins, which are involved in embryonic and somatic stem cell function, embryonic development, hepatocyte membrane transport and growth control, have been demonstrated a relative lack of sensitivity and specificity for identifying liver CSCs [10-12]. So far, no markers for putative liver CSCs have yet been generally accepted, and further study is needed to explore the isolation method for liver CSCs.

A major advance in adult stem cell research was achieved in 1996 when it was discovered that the undifferentiated multipotent neural cells could be grown and maintained in suspension using the neurosphere assay [13]. Then anchorage-independent sphere culture of stem cells was instrumental in the study of adult stem cells including the nerve, prostate and mammary stem cells [14-17]. Recently, as a functional approach, sphere formation is particularly useful to enrich the potential CSC subpopulations when the specific CSC makers have not been defined as the case for most CSCs [18-25]. However, there have been few reports for sphere culture in liver cancer. Therefore, the present study intends to establish an alternate approach to isolate, identify and characterize liver cancer cell subsets with CSC properties.

## Methods

### Cell lines and sphere culture

Human hepatoma cell lines, PLC/PRF/5 and HepG2, were obtained from the Cell Bank of Chinese Academy of Sciences (Shanghai, China). MHCC97H was obtained from the Liver Cancer Institute, Zhongshan Hospital, Fudan University (Shanghai, China). All of the cells were maintained as a monolayer in high glucose DMEM with 10% fetal bovine serum (FBS), 100 IU/ml penicillin G and 100 μg/ml streptomycin at 37°C in a humidified 5% CO_2 _incubator. Cells were collected and washed to remove serum, then suspended in serum-free DMEM/F12 supplemented with 100 IU/ml penicillin, 100 μg/ml streptomycin, 20 ng/ml human recombinant epidermal growth factor (hrEGF), 10 ng/ml human recombinant basic fibroblast growth factor (hrbFGF), 2% B27 supplement without vitamin A, 1% N2 supplement (Invitrogen, Carlsbad, CA, USA). The cells were subsequently cultured in ultra low attachment 6-well plates (Corning Inc., Corning, NY, USA) at a density of no more than 5,000 cells/well.

### Sphere passage and sphere formation assay

The spheres were collected by gentle centrifugation, then dissociated with trypsin-EDTA and mechanically disrupted with a pipette. The resulting single cells were then centrifuged to remove the enzyme and re-suspended in serum-free medium allowed to re-form spheres. The spheres should be passaged every 5-8 days before they reached a diameter of 100 μm. The dissociated single sphere-forming cells were also diluted to a density of 500 cells/ml. Then, the 2 μl/well diluted cell suspension was plated to ultra low attachment 96-well plate (Corning Inc., Corning, NY, USA), and 150 μl of serum-free medium was added. The wells with only one cell were marked and observed everyday.

### Colony formation assay

The PLC/PRF/5 spheres were enzymatically dissociated as described above. Trypan blue staining was used to determine cell viability, and more than 95% of cells with viability were acceptable for the following experiments. The single cells were seeded in DMEM with 10% FBS at a density of 2000 cells/well on 6-well plates that were pre-coated with Matrigel (BD Biosciences, San Jose, CA, USA). After 7 days, the colony formation ability was assessed by counting the number of colonies (> 70 cells) under a microscope after crystal violet staining (Sigma-Aldrich, St. Louis, MO, USA). Representative views were photographed. The parental cells were plated at the same density as the control.

### Chemotherapy sensitivity assays

The sensitivity of the PLC/PRF/5 parental and sphere-forming cells to chemotherapeutic drugs was measured by MTT assay. Briefly, cells were seeded in 96-well plates that were precoated with Matrigel, and various concentrations of cisplatin (Sigma-Aldrich) were added at the beginning, co-incubated for 12 h or 24 h. After changing to fresh medium without cisplatin, cells were cultured for another 72 h. The MTT reagent (Sigma-Aldrich) was then added to each well according to the manufacturer's instructions. Absorbance was measured at 490 nm.

### Immunofluorescent staining

Cells were fixed in 4% paraformaldehyde and blocked with normal goat serum. The primary antibodies, including mouse anti-human OV6 (R&D Systems Inc., Minneapolis, MN, USA), mouse anti-human CD133 and rabbit anti-human CD44 (Santa Cruz Biotechnology, Inc. Santa Cruz, CA. USA) were added and incubated overnight at 4°C. After washing 3 times with PBS, the goat anti-mouse IgG and goat anti-rabbit secondary antibodies conjugated with Cy3, FITC or TRITC (Jackson ImmunoResearch Laboratories Inc., West Grove, PA, USA) were added and incubated at room temperature for 1 h. Cells were then counterstained with DAPI (Sigma-Aldrich) and the images were captured using an Olympus-IX71 fluorescent microscope (Olympus Inc., Center Valley, PA, USA).

### In vivo tumorigenicity experiments

All mice were cared for in accordance with institutional guidelines. The PLC/PRF/5 parental and the third, sixth and ninth passages of sphere-forming cells were used in tumorigenicity experiments. Trypan blue staining was used to assess cell viability, and various numbers of viable single cells were subcutaneously injected into 5-week-old NOD/SCID male mice (Shanghai Laboratory Animal Center, Chinese Academy of Sciences, Shanghai, China) in serum-free DMEM/Matrigel (1:1) using 100 μl microsyringe. Mice were killed at 8 weeks after cell injection, then the tumors were harvested for further examination.

### Real-time PCR microarray analysis

Human Stem Cell RT^2 ^Profiler™ PCR Array (PAHS-405A, SABioscience, USA) was done according to the manufacturer's instructions. Briefly, RNA samples from both the PLC/PRF/5 parental cells (as control) and the tertiary passage spheres were prepared. After removal of contaminating DNA from RNA preparations, total RNA samples were cleaned up and the resulting RNA were assessed for both yield and quality. After the first strand cDNA was synthesized, real-time PCR was performed. The data were analyzed using the ΔΔCt method.

### Western blotting analysis

Quantified protein lysates were resolved on SDS-PAGE gels, transferred onto a polyvinylidene difluoride membrane (Millipore, Billerica, MA, USA), and immunoblotted with the primary antibodies against EpCAM, CD133, activated Notch1, Ep300 (Abcam, Cambridge, MA, USA), DTX1, Oct3/4 (Santa Cruz, CA, USA), or OV6, CD44 (R&D Systems Inc., MN, USA), followed by incubation with the horseradish peroxidase-conjugated secondary antibody. The blots were visualized using a supersignal west femto maximum sensitivity substrate kit (Pierce, Waltham, MA, USA). GAPDH was used as a loading control.

### Statistical analysis

All values in the figures and text were showed as means ± SD. Statistical analyses were performed using the SPSS statistical software package (SPSS/PC+, SPSS Inc., Chicago, IL, USA). Any significant differences among mean values were evaluated by the student's *t *test. A two-sided *P *< 0.05 was accepted as significant.

## Results

### Hepatoma cells could form anchorage-independent, self-renewing spheres

**Figure 1:**
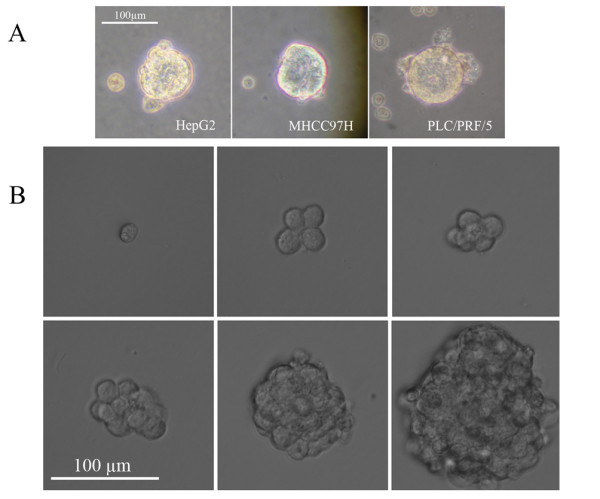
**Hepatoma cells formed the anchorage-independent, self-renewing spheres**. (A) Hepatoma cell lines, including HepG2, MHCC97H and PLC/PRF/5, could form the anchorage-independent 3-D spheres in stem cell conditioned culture medium (200×). (B) Generation of a sphere from a single PLC/PRF/5 cell. The propagation of a single cell cultured in a 96-well dish was recorded at day 1, 3, 5, 7, 9 and 13, separately (400×).

The hepatoma cells were plated in stem cell conditioned culture medium in 6-well plates at a density of 5,000 cells/well which allowed for the formation of colonies separated from each other. In this condition, cells grew as nonadherent, three-dimensional sphere clusters, called spheres. Figure 1A showed anchorage-independent spheres formed by the HepG2, MHCC97H and PLC/PRF/5 cells. After 5 to 8 days, when the spheres grew to 70 to 100 μm in diameter, they were passaged and the single cell from spheres could propagate to form new spheres again. A key property of all normal and cancer stem cells is their unique ability to self-renew. One of the methods to determine the self-renewal capacity of sphere-forming cells is to test their capability of serial passage. The PLC/PRF/5, HepG2 and MHCC97H spheres had been serially passaged for more than 12 generations, indicating their self-renewal capability in vitro.

To corroborate the finding that a sphere could be generated from a single cell, one PLC/PRF/5 cell per well was plated to a 96-well plate and the wells with one cell were visualized everyday. Figure 1B showed the process of single PLC/PRF/5 cell forming a sphere.

### Sphere-forming cells proliferate extensively in vitro

**Figure 2:**
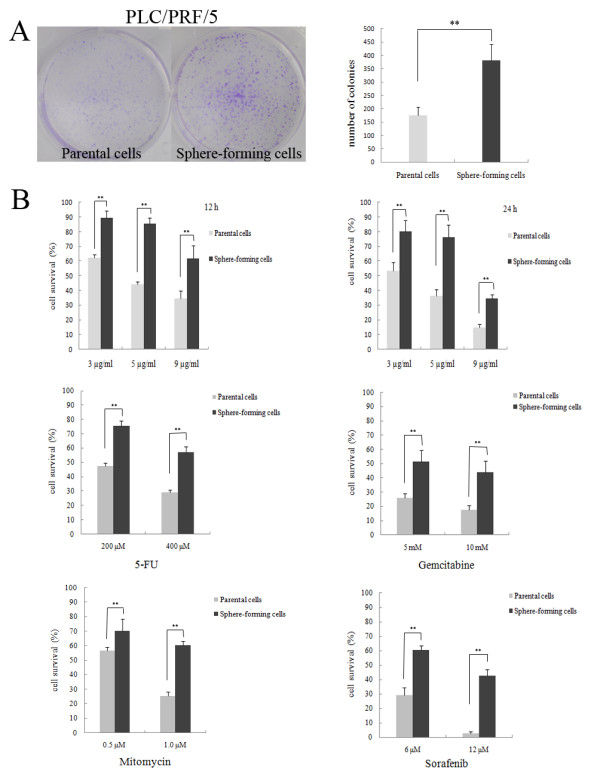
**Sphere-forming cells proliferated extensively and possessed resistance ability to conventional chemotherapeutics in vitro**. (A) Comparison of in vitro clonogenecity of the PLC/PRF/5 parental cells and sphere-forming cells. Cells were plated at a density of 2,000 cells/well in 6-well plates and cultured for 1 week. At the end, cells were stained with crystal violet, photographed, and analyzed for their proliferation efficiency. Each experiment was performed in triplicate, and the representative examples were shown (Columns, mean; bars, SD; *, *P *< 0.01). (B) The PLC/PRF/5 sphere-forming cells showed a drug resistance phenotype. The parental cells and sphere-forming cells were treated with cisplatin at the beginning of plating for 12 h or 24 h and 5-Fu, gemcitabine, mitomycin and sorafenib for 36 h. Cell survival was determined by MTT assay (**, *P *< 0.01).

We compared the proliferative ability of the tertiary passage PLC/PRF/5 spheres and its parental cell line using clonogenicity assay. Both of them were adherently plated and were alive, but were unable to form comparable colonies. The sphere-forming cells proliferated significantly faster and induced bigger and greater numbers of tumor colonies than the parental cells. Based on counting the number of colonies per 2,000 seeded cells, it was 175.67 ± 30.07/381.00 ± 61.02 (*P *= 0.006) for the parental/sphere-forming PLC/PRF/5 cells (Figure 2A). The sphere-forming cells were capable of extensive proliferation, indicating that the sphere-forming cells could play an important role in the maintenance of tumor growth.

### Sphere-forming cells possess the ability of resistance to conventional chemotherapy in vitro

HCC cells are commonly resilient to chemotherapy. It is speculated that cancer stem or progenitor cells in HCC are more resistant to conventional chemotherapy contributing to recurrence. To examine whether the self-renewing sphere-forming cells possess a hypothesized CSC chemoresistant property, the sensitivity of the PLC/PRF/5 parental cells versus the tertiary passage sphere-forming cells to cisplatin was assessed. The survival rates of sphere-forming cells were higher under the treatment of 3 μg/ml, 5 μg/ml and 9 μg/ml cisplatin for 12 h, being 1.4-fold, 1.9-fold, 1.8-fold, respectively, compared with the parental PLC/PRF/5 cells (*P *< 0.01), whereas, under the treatment for 24 h, the relative survival rates were increased to 1.5-fold, 2.1-fold and 2.3-fold respectively (Figure 2B, P < 0.01).

We also tested the sensitivity of sphere-forming cells to other 4 drugs in addition to cisplatin. The PLC/PRF/5 sphere-forming cells exhibited general resistance to 5-Fu, gemcitabine, mitomycin and sorafenib in the treatment of 36 h. Compared with the PLC/PRF/5 parental cells, the survival rates of PLC/PRF/5 sphere-forming cells were higher under 200 μmol/L, 400 μmol/L of 5-Fu (1.60-fold, 1.98-fold respectively, *P *< 0.01); 5 mmol/L, 10 mmol/L of gemcitabine (1.99-fold, 2.49-fold respectively, *P *< 0.01); 0.5 μmol/L, 1.0 μmol/L of mitomycin (1.24-fold, 2.33-fold respectively, *P *< 0.01); and 6 μmol/L, 12 μmol/L of serafenib (2.07-fold, 15.21-fold respectively, *P *< 0.01). The results support a role for these sphere-forming cells in HCC chemoresistance, which may explain why current therapies fail to eradicate progenitors and prevent tumor re-growth.

### Sphere-forming cells exhibit high tumorigenicity in vivo

**Figure 3:**
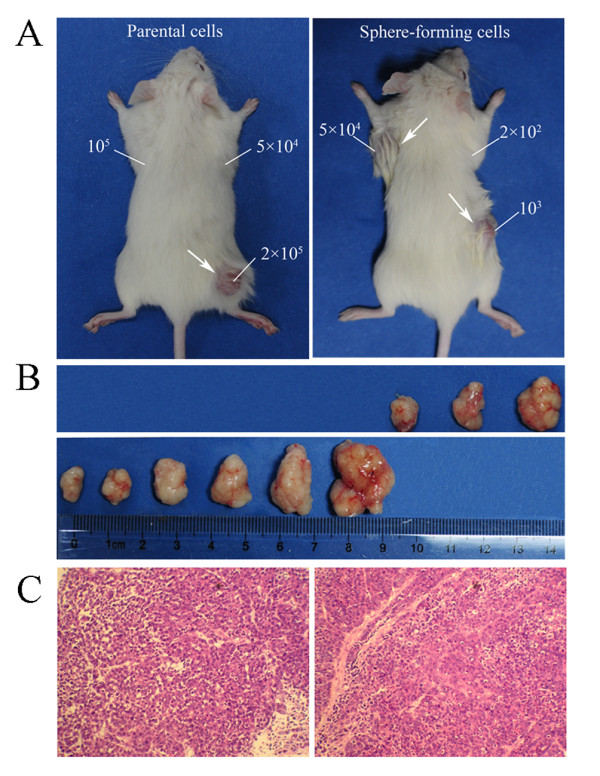
**Sphere-forming cells exhibited high tumorigenicity in vivo**. (A) The representative examples of xenograft tumors formed after subcutaneous injection with the PLC/PRF/5 parental cells and sphere-forming cells. (B) The top row shows the nodules formed by injecting 2 × 10^5^, 5 × 10^5 ^and 1 × 10^6 ^PLC/PRF/5 parental cells, separately. The bottom row shows the nodules formed by injecting 5 × 10^2^, 1 × 10^3^, 2.5 × 10^3^, 5 × 10^3^, 1 × 10^4 ^and 5 × 10^4 ^PLC/PRF/5 sphere-forming cells, separately. (C) H&E staining revealed that the histological features of xenograft tumors induced by the PLC/PRF/5 sphere-forming cells were similar to those induced by the parental cells.

To confirm that the sphere-forming cells exhibit greater tumor initiating capability, NOD/SCID mice were transplanted with varying amounts of the PLC/PRF/5 sphere-forming cells ranging from the amount that is unable to initiate tumor growth to the amount that always initiates tumor formation. The PLC/PRF/5 parental cells were operated as controls. As few as 500 sphere-forming cells were sufficient for tumor development, whereas, at least 2 × 10^5 ^parental cells were necessary to consistently generate a tumor in the same model, and not to mention, requiring a longer period of time (Table 1 Figure 3). The tumor nodules formed by the PLC/PRF/5 sphere-forming cells displayed similar histology to that by the parental cells. To inspect whether the tumor initiating capability could be decreased as the spheres were passaged, we also compared the tumorigenicity of different generations of spheres. The results showed that both the tumor initiating capability and phenotypic appearance were similar for the 3th, 6th and 9th generations of sphere-forming cells (Table 1). The tumorigenic efficacies of three cell lines HepG2, PLC/PRF/5 and MHCC97H were also compared in nude mice (Additional file 1 Figure S1). The results suggested that the tumorigenic efficacies of sphere-forming cancer cells were enhanced compared with the parental cells, and the volumes of tumors were positively correlated with malignant grade of the cell lines (malignant grade HepG2 < PLC/PRF/5 < MHCC97H). Interestingly, the HepG2 parental cells at 10^6 ^cells/mouse could not form visible xenografts (0/5), but the HepG2 sphere-forming cells at 10^6 ^cells/mouse could form xenograft tumors in the same period of 30 days.

**Table 1:** Tumorigenicity experiments of PLC/PRF/5 sphere-forming cells and parental cells in NOD/SCID mice

Cell type	Cell numbers injected	Tumor incidence^†^	Latency(days)^‡^
Sphere-forming cells of the 3rd generation	2 × 10^2^	0/3	-
	5 × 10^2^	1/3	35
	1 × 10^3^	3/3	23
	2.5 × 10^3^	3/3	23
	5 × 10^3^	3/3	20
	1 × 10^4^	3/3	16
	5 × 10^4^	2/2	9
	1 × 10^5^	2/2	7
	2 × 10^5^	2/2	7

Sphere-forming cells of the 6th generation	2 × 10^2^	0/3	-
	5 × 10^2^	2/3	49
	1 × 10^3^	3/3	28

Sphere-forming cells of the 9th generation	2 × 10^2^	0/3	-
	5 × 10^2^	1/3	40
	1 × 10^3^	3/3	30

PLC/PRF/5 parental cells	5 × 10^4^	0/3	-
	1 × 10^5^	0/3	-
	2 × 10^5^	2/3	30
	5 × 10^5^	3/3	16
	1 × 10^6^	2/2	9

^†^The number of tumors detected/number of injections.

^‡^Approximate number of days from tumor cell injection to appearance of a tumor.

### Sphere-forming cells overexpress liver CSC related proteins and the CSL-independent Notch signaling pathway might play a role in liver CSCs

**Figure 4:**
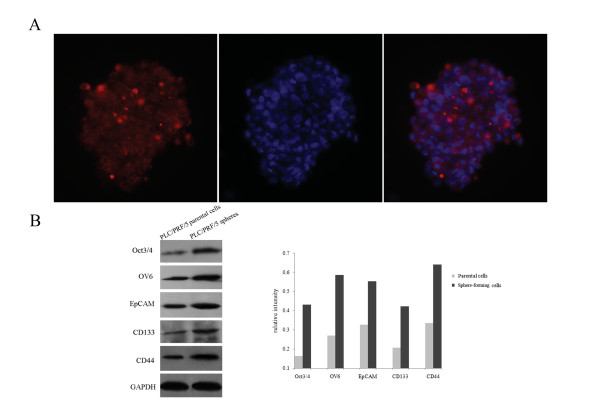
**Sphere-forming cells overexpressed liver CSC related proteins and downstream proteins of the CSL-independent Notch pathway**. (A) The PLC/PRF/5 sphere-forming cells expressed the hepatic stem cell maker OV6, as observed under fluorescence microscopy. Nuclei were stained with DAPI (400×). (B) Western blotting assay showed the liver CSC related proteins (Oct3/4, OV6, EpCAM, CD133 and CD44) and the relative band intensities were calculated by densitometry and normalized to the loading control GAPDH.

To date, anti-OV6, a monoclonal antibody raised against cells isolated from carcinogen treated rat liver [26], remains the best available marker of hepatic stem cells [27], even though it also reacts with bile duct epithelium in rats and humans [28]. Besides, Yang *et al *reported that the OV6^+ ^liver cancer cells may represent a potential stem/progenitor-like cell population [8]. Immunofluorescent staining for OV6 showed that most of the PLC/PRF/5 sphere-forming cells are OV6 positive (Figure 4A). Then, liver CSCs related proteins, including Oct3/4, OV6, EpCAM, CD133 and CD44 were examined by Western blotting. The results displayed that all of them were significantly increased as compared to the parental cells (Figure 4B), denoting that the PLC/PRF/5 spheres possess stem cell-like properties. We also compared the expression of candidate CSC markers CD133 and CD44 between the parental and sphere-forming cells of HepG2 and MHCC97H by immunofluorescent labelling. It was found that CD44 expression was obviously enriched in HepG2 and MHCC97H sphere-forming cells compared with their parental cells (Additional file 2, Figure S2).

**Figure 5:**
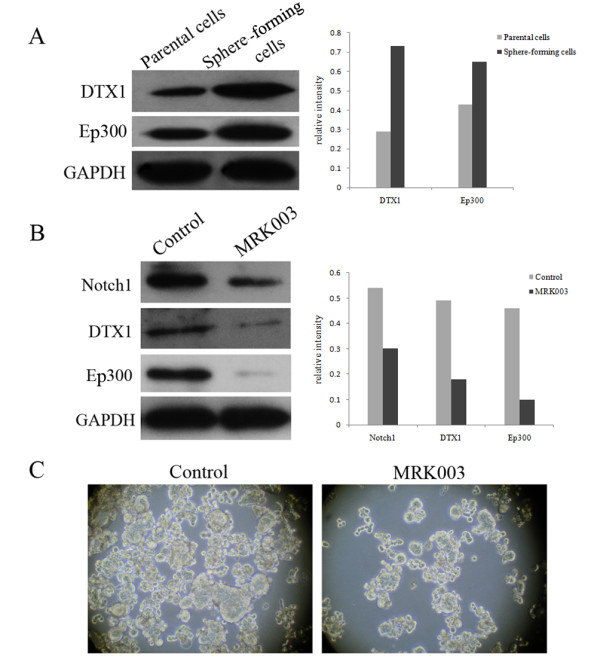
**CSL-independent Notch signaling pathway might play a role in liver CSCs and MRK003 could partly eliminate the stem-like cells**. (A) The downstream proteins levels of the CSL-independent Notch pathway (DTX1 and Ep300) were significantly increased in the PLC/PRF/5 spheres, compared with the parental cell line. GAPDH was used as a loading control. (B) 10 μM MRK003 could reduce the activation of Notch1 and downstream components of CSL-independent Notch signaling pathway DTX1 and Ep300 were consequently decreased. (C) The secondary dissociated PLC/PRF/5 sphere-forming cells were treated with 10 μM MRK003 or DMSO control for 7 days. The sphere formation ability of the MRK003-treated groups were significantly inhibited in comparison to DMSO-treated controls.

To further explore the biological properties of liver CSCs, the Stem Cell RT^2 ^Profiler™ PCR array was performed. This array profiles the expression of 84 genes related to the identification, growth and differentiation of stem cells. Among the significantly distinguishing genes, we noted the DTX1 and Ep300, CSL-independent Notch signaling pathway related genes, were 4.24-fold and 2.36-fold, respectively, more abundant in the spheres than those in the control. Consistent results were confirmed by Western blotting (Figure 5A). To determine the role of CSL-independent Notch signals, blocking Notch pathway was performed by a gamma-secretase inhibitor MRK003. The secondary dissociated PLC/PRF/5 sphere-forming cells were treated with 10 μM MRK003 or DMSO control for 7 days. The inactivation of Notch1 and down-regulations of downstream target genes DTX1 and Ep300 were confirmed by Western blotting (Figure 5B). The sphere formation ability of the MRK003-treated groups was significantly inhibited in comparison to the DMSO-treated controls (Figure 5C). The results indicated that the CSL-independent Notch signaling pathway might play an important role in liver CSCs and MRK003 could partly eliminate the stem-like cells.

## Discussion

There are two classical models of carcinogenesis [29,30]. One is the stochastic model, which is based on the belief that most of tumor cells are capable of extensive proliferation and contribute substantially to tumor maintenance; carcinogenesis could results from the random mutations and the subsequent clonal selections. The other is the hierarchical model, which is based on the belief that there are hierarchical differences among tumor cells, and only a small number of specific cells capable of extensive proliferation can contribute to carcinogenesis. The discovery of CSCs in solid tumors strongly supports the hierarchical model. The CSC hypothesis considers that CSCs subsets are located in the top of the hierarchical structure of tumor cells and directly affect the organization and construction of lower hierarchical cells. Therefore, the identification of tumorigenic liver CSCs could provide new insight into the HCC tumorigenic process and possibly bear great therapeutic implications.

Usually, the isolation and identification of liver CSCs involve two types of methods: one is based on the sorting of side population (SP) cells that can exclude the hoechst 33342 dye [31]. However, Hoechst 33342 is cytotoxic; consequently, SP cells are protected by their membrane transport properties, whereas unprotected non-SP cells suffer toxicity and are unable to grow. Thus the differing tumor-initiation abilities of SP and non-SP cells are most likely due to an artifact of Hoechst 33342 toxicity, rather than due to intrinsic stem-cell properties [32]. The other type includes the fluorescence activated cell sorting (FACS) and the magnetic activated cell sorting (MACS), which are based on cell surface markers. The proposed markers for liver CSCs include CD133, CD90, CD44, CD13, EpCAM and OV6, on the basis of the hypothesis that CSCs are originated from somatic stem cells and accordingly express the same surface markers [33,34]. Although they have been reported to be used to enrich CSC fraction, their sensitivity and specificity for identifying liver CSCs are being challenged. For example, Kimura *et al *reported CD133^+ ^fraction in Hep3B and Huh7 were 16.8% and 2.7%, respectively [7], whereas some other groups reported more than 90% in Hep3B and 60% in Huh7 [5,6]. In Huh7 the CD13-positive cells typically existed in a CD133^strong ^fraction, but in PLC/PRF/5 the CD13-positive cells were CD133-negative [9]. Some researchers indicated that different culture conditions and differentiated degree of the cells, especially the latter, were important factors. The roles of these phenotypes in defining functionally distinct populations of cells from progenitor to differentiated hepatocytes need to be systemically studied.

Recently, sphere culture has been increasingly used as a method for enriching stem cells which relies on their property of anchorage independent growth. Researchers have reported the application of sphere culture to isolate, enrich, maintain or expand potential CSC subpopulations from various types of cancers [18-25]. The sphere-forming cells from primary tumors, such as breast cancer and ovarian cancer, showed stem-like properties and expressed their CSC markers [19,23]. It is generally agreed that, like all stem cells, the tumor sphere-forming cells are capable of proliferation, self-renewal and possess higher tumorigenicity. Using neural crest stem cell conditions and sphere formation system, Hansford *et al *[35] for the first time successfully expanded tumor cells both from low-risk neuroblastomas and from the bone marrow metastases of high-risk tumors. The latter formed metastatic tumors in a murine xenograft model with as few as 10 cells and could also be serially passaged [35]. To our knowledge, there have been few reports on the isolation and long-term propagation of liver CSCs by the method of sphere culture.

In the present study, using stem cell conditioned culture system, we tested three human hepatoma cell lines, PLC/PRF/5, MHCC97H and HepG2. Cells are plated at a low density (< 5000 cells/well in 6-well plate) to avoid spontaneous cell aggregation. The three cell lines could form clonal nonadherent 3-D spheres, and without exceptions, could also be serially passaged. We evaluated the PLC/PRF/5 sphere-forming cells for their stemness characteristics. It has been showed that they were capable of self-renewal, proliferation, drug resistance, and overexpressing liver CSC related proteins. Xenotransplantation is the gold standard for evaluating tumorigenicity of tumor cells. We tested the third, sixth and ninth generations of the PLC/PRF/5 sphere-forming cells for their tumor initiating capability. It was demonstrated that as few as 500 cells from the PLC/PRF/5 spheres were able to form a tumor when subcutaneously injected into NOD/SCID mice, while 2 × 10^5 ^parental cells were needed. This was 400 times higher than that of sphere-forming cells. Moreover, the tumor initiating capability was not decreased as the spheres were passaged. Similar CSC properties of self-renewal, strongly proliferation, drug resistance and tumorigenicity are also found in the MHCC97H and HepG2 spheres. Indeed, the HepG2 parental cells at 10^6 ^cells/mouse could not form visible xenografts in nude mice, but its sphere-forming cells at the same amount of cells could form xenograft tumors, suggesting the tumorigenic efficacies of sphere-forming cells were enhanced compared with the parental cancer cells.

To further explore the CSC properties of sphere-forming cells, we examined the sensitivity of sphere-forming cells to chemotherapeutics and the expression of candidate CSC markers. The PLC/PRF/5 sphere-forming cells exhibited general resistance to cisplatin, 5-Fu, gemcitabine, mitomycin and sorafenib, and showed higher survival percentages compared with its parental cells. Synchronously, we found that CD44 expression was obviously enriched in HepG2 and MHCC97H sphere-forming cells compared with their parental cells. CD44 is a polymorphic family of immunologically related cell surface proteoglycans and glycoproteins, normally takes part in cell-cell and cell-matrix adhesion interactions, which is involved in cancer cell migration, proliferation and metastasis. Accordingly, CD44 expression enrichment in sphere-forming cells may account for their increased survival ability and tumorigenicity. Therefore, we propose that the nonadherent tumor spheres cultured in serum-free condition possess liver CSC properties. This long-term culture system may also provide the means of further purifying and functionally characterizing the biological properties of the liver CSC fraction, with the goal of developing new therapeutic strategies directed specifically against liver CSCs.

Accumulating evidence has been established that the Notch signaling pathway plays vital and universal roles not only in cell differentiation, embryonic development and tissue self-renewal, but also in pathogenesis of some types of human cancers and genetic disorders. Recent advancements have further revealed that the Notch signals produce a marked effect either in stem cells or CSCs. The activated Notch signals can inhibit hematopoietic stem cell differentiation and maintain their pluripotency [36,37], and maintaining the stem cell population in several solid tissue types, including several neuroectodermal tissues [38]. Only when the Notch signals are activated the cancer stem cell activity could be enhanced to promote intestinal tumor formation [39]. Generally, the Notch signaling pathway is mediated in two different pathways. One is through CSL-DNA binding proteins; the other is the CSL-independent pathway. DTX1 (Deltex-1) is an important transcriptional regulator that is downstream of the Notch receptor in the CSL-independent Notch signaling pathway [40]. Ep300, also known as p300, is a transcriptional co-activator protein. It functions as a histone acetyltransferase that regulates transcription via chromatin remodeling and is important in the processes of cell proliferation and differentiation. It has been reported that Ep300 can work as a transcriptional co-activator of DTX1. Yamamoto *et al *[41] reported that DTX1 inhibited the transcriptional activation of the neural-specific helix-loop-helix type transcription factor MASH1 by binding to Ep300. This mechanism is likely responsible for the differentiation inhibition of neural progenitor cells. In our study, the result of stem cell microarrays showed that DTX1 and Ep300 were highly expressed in liver cancer stem-like cells. This was further confirmed by Western blotting. Although the molecular mechanism and function of the CSL-independent Notch signaling pathway have not been elucidated, and little has been known about its involvement in HCC, we suppose that the CSL-independent Notch signaling pathway play an important role in the differentiation and propagation of liver CSCs.

## Conclusions

In summary, the study demonstrated that the nonadherent tumor spheres from human hepatoma cell lines which are cultured in stem cell conditioned medium possess liver cancer stem cell properties, and the CSL-independent Notch signaling pathway may play a role in the differentiation and propagation of liver CSCs.

## Competing interests

The authors declare that they have no competing interersts.

## Authors' contributions

Lu C conceived the study and participated in the study design, performance, coordination and manuscript writing. YMZ participated in the study performance and writing. MCW participated in the study design and coordination. ZFY participated in the study design, coordination and writing. BBZ, Jian L, WX, RXZ, Jing L, YZ, Lei C and HHQ performed the research. All authors have read and approved the final manuscript.

## Pre-publication history

The pre-publication history for this paper can be accessed here:

http://www.biomedcentral.com/1471-230X/11/71/prepub

## Supplementary Material

Additional file 1 **Figure S1**. Tumorigenic efficacies of the hepatoma sphere-forming cells. The hepatoma parental cells and sphere-forming cells were injected into nude mice subcutaneously at the indicated cell concentrations. At day 30^th ^after injection, mice were sacrificed and tumors were removed and compared in size. Click here for file

Additional file 2 **Figure S2**. Expression of candidate CSC markers in hepatoma sphere-forming cells. Confocal immunofluorescent staining showed that CD44 expression was enriched obviously in HepG2 and MHCC97H sphere-forming cells compared with their parental cells. Nuclei were stained with DAPI (400×). Click here for file
