# Supplementary figures and images for: Sphere-forming cell subpopulations with cancer stem cell properties in human hepatoma cell lines
Source: BMC Gastroenterol. 2011 Jun 14;11:71. doi: 10.1186/1471-230X-11-71 (PMC3136412; doi:10.1186/1471-230X-11-71)

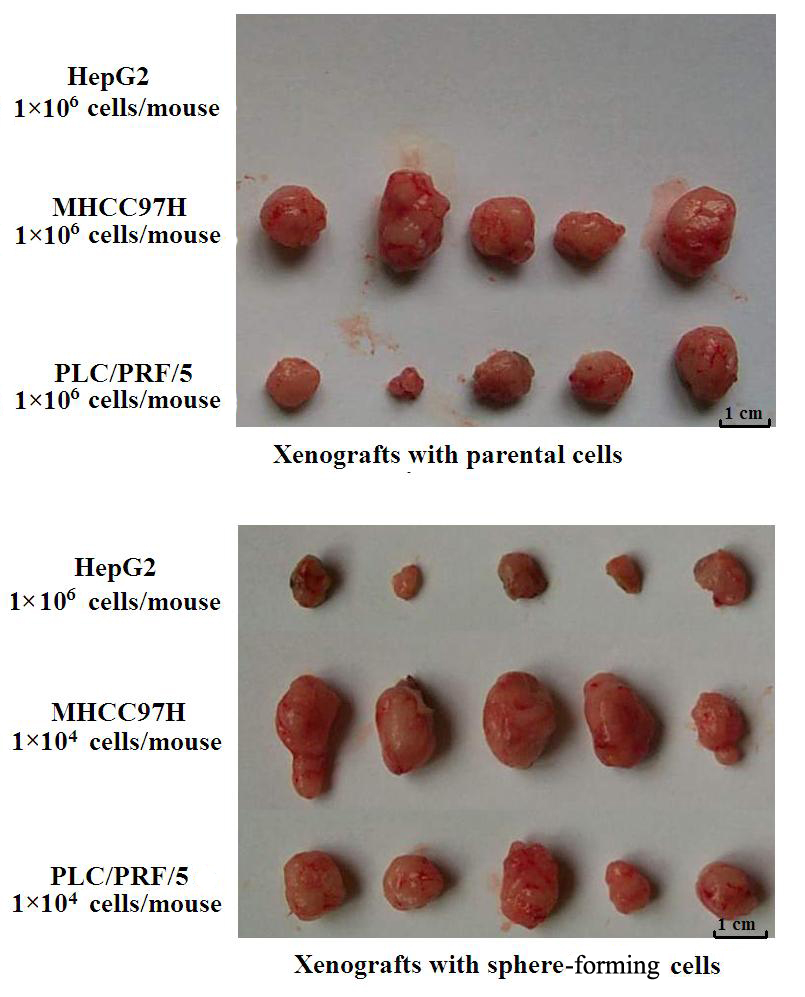

Supplement: Additional file 1 — Figure S1. Tumorigenic efficacies of the hepatoma sphere-forming cells. The hepatoma parental cells and sphere-forming cells were injected into nude mice subcutaneously at the indicated cell concentrations. At day 30th after injection, mice were sacrificed and tumors were removed and compared in size. [file 1471-230X-11-71-S1.TIFF]

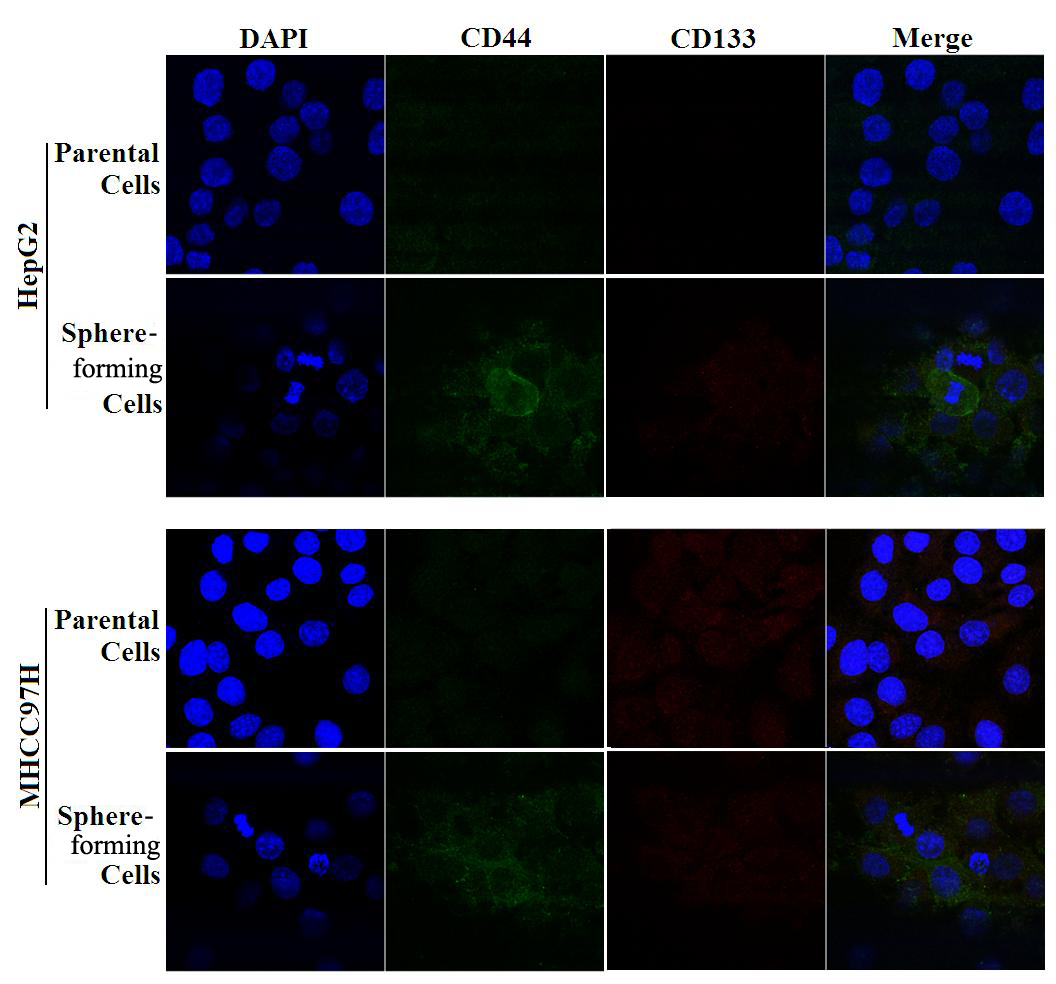

Supplement: Additional file 2 — Figure S2. Expression of candidate CSC markers in hepatoma sphere-forming cells. Confocal immunofluorescent staining showed that CD44 expression was enriched obviously in HepG2 and MHCC97H sphere-forming cells compared with their parental cells. Nuclei were stained with DAPI (400×). [file 1471-230X-11-71-S2.TIFF]
